# Mark4 Inhibited the Browning of White Adipose Tissue by Promoting Adipocytes Autophagy in Mice

**DOI:** 10.3390/ijms21082752

**Published:** 2020-04-15

**Authors:** Kun Yang, Jiarui Cai, Miao Pan, Qian Sun, Chao Sun

**Affiliations:** Key Laboratory of Animal Genetics, Breeding and Reproduction of Shaanxi Province, College of Animal Science and Technology, Northwest A&F University, Yangling 712100, Shaanxi, China; kunyang424@gmail.com (K.Y.); jiaruicai412@gmail.com (J.C.); miaopan341@gmail.com (M.P.); qiansun724@gmail.com (Q.S.)

**Keywords:** *Mark4*, autophagy, browning of white adipose tissue

## Abstract

Autophagy can remove excess or dysfunctional proteins and organelles to maintain cellular homeostasis. Browning of white adipose tissue increases the energy expenditure. Microtubules affinity regulated kinase 4 (Mark4) can regulate a variety of physiological processes. According to previous studies, we speculated that Mark4-autophagy-browning of white adipose tissue had certain linkages. Here, we established two autophagy models through serum starvation and rapamycin treatment and detected that the overexpression of *Mark4* increased the expression of autophagy-related factors Beclin1, ATG7, and significantly decreased the autophagy substrate P62. Further tests showed that the overexpression of *Mark4* promoted the conversion of autophagy marker protein LC3A to LC3B-II by activating the AMP-activated protein kinase (AMPK) pathway and inhibition of the AKT/mTOR signaling. Moreover, *Mark4* decreased the expression of thermogenesis genes via promoting autophagy. These results indicated that *Mark4* inhibited the browning of white adipose tissue via promoting autophagy.

## 1. Introduction

MAP/MARK4 belongs to the cell microtubule associated protein (MAPs) serine/threonine kinase (MARKs) family and involves in a variety of physiological processes [1]. The *Mark4* gene encodes two alternate splicing isoforms, including L-type and S-type which differ in the C-terminal region [2]. *Mark4L* is highly expressed in the brain and may be involved in the development of the nervous system and the differentiation of neurons, while *Mark4S* is highly expressed in tumor cells and glioma cells, indicating that it may be involved in the regulation of cell cycle [3,4,5,6]. Studies indicate that *Mark4* is the negative regulator of mTORC1 which plays a central role in cell growth [7,8]. Recently, the researchers found that *Mark4* knockout mice increased their appetite, activity, and metabolic rate to resist obesity caused by a high-fat diet. At the same time, the protein kinase B (PKB/AKT) and AMP-activated protein kinase (AMPK) signaling pathways were activated to reduce insulin resistance and maintain glucose homeostasis [9]. Further research determined that *Mark4* promoted adipogenesis by activating Jun N-terminal kinase (JNK) and p38-mitogen-activated protein kinase (MAPK) signaling pathways, it also triggered adipocytes apoptosis by activating the JNK signaling pathway [10]. Additionally, studies have found that *Mark4* promoted oxidative stress and inflammation via binding to the peroxisome proliferator-activated receptor gamma (PPARγ) and activating nuclear factor-kappa B (NF-κB) pathway in mice adipocytes. These data establish a novel regulation role of Mark4 on body metabolic balance [11].

Autophagy is an evolutionarily conserved lysosome-dependent system in eukaryotes that transports cytosolic components to the lytic compartment of the cell for degradation [12,13]. Previous studies have found that nutrient deprivation-induced loss of lipid droplets was related to autophagy, whereas the inhibition of autophagy increased the storage of triglycerides in lipid droplets [14]. Knockdown of autophagy-related gene 7 (*Atg7*) in 3T3-L1 preadipocytes inhibits lipid accumulation and decreases protein levels of adipocyte differentiation factors. The adipocyte-specific knockout of *Atg7* generates enhancement of insulin sensitivity and the features of brown adipocytes in mouse white adipose tissue, resulting in the lowering of white adipose mass [15]. Autophagy is the most active and essential for the initial stage of adipocyte differentiation, but it is dispensable during its later stage [16]. Studies have found that mineralocorticoid receptor (MR) antagonism induced browning of white adipose tissue through impairing autophagy and preventing adipocyte dysfunction in high-fat diet fed (HFD) mice [17]. A research found that natural plant alkaloid-berberine inhibited basal autophagy in adipocytes and adipose tissue of mice fed a high-fat diet via downregulating expression of *Beclin1* [18]. Mice with skeletal muscle-specific deletion of *Atg7* have a reduced fat content and been protected from diet-induced obesity and insulin resistance. This phenotype is accompanied by increased fatty acid oxidation and browning of white adipose tissue (WAT) owing to the induction of fibroblast growth factor 21 (Fgf21) [19]. Our previous research found that leptin inhibited ER stress-induced inflammation through reducing Activating transcription factor 4 (Atf4)-autophagy-related gene5 (*Atg5*)-mediated autophagy in adipocytes [20].

At present, the relationship among Mark4, autophagy, and browning of white adipose tissue has not been reported in adipose tissue [21,22]. Here, we demonstrated that Mark4 promoted adipocytes autophagy which was induced by serum starvation and rapamycin treatment. Furthermore, we found that the browning of white adipose tissue was inhibited via promoting autophagy. These finding elucidated a novel function of Mark4 in the regulation of cell autophagy and browning of white adipose tissue, implying a potential strategy for metabolism syndrome 

## 2. Results

### 2.1. Serum Starvation and Rapamycin Treatment can Induce Adipocyte Autophagy

**Figure 1:**
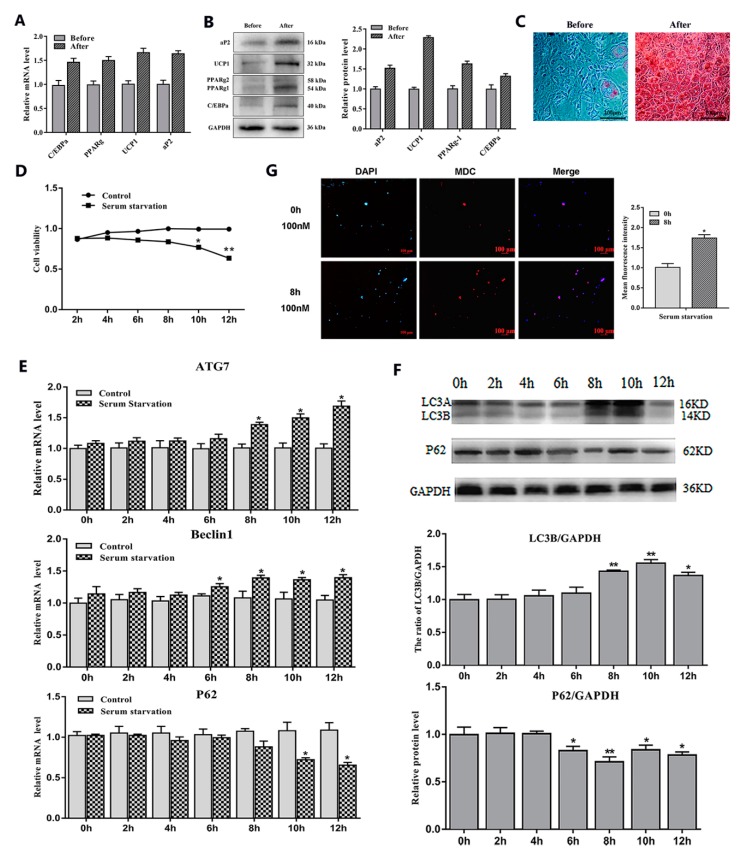
Adipocytes autophagy was induced by serum starvation treatment. (**A**) Relative mRNA level of adipogenic differentiation-related genes (*n* = 6). (**B**) Protein level of adipogenic differentiation-related genes (*n* = 6). (**C**) Representative images of differentiated cells were labeled with Oil Red O (*n* = 6). (**D**) Cell viability was detected by bycholecystokinin-8 (CCK8) (*n* = 6). (**E**) Relative mRNA level of autophagy-related genes with or without serum starvation for different lengths of time (*n* = 6). (**F**) Protein level of autophagy-related genes with or without serum starvation for different lengths of time (*n* = 6). (**G**) Monodansylcadaverine (MDC) and DAPI staining by serum starvation treatment for 8 h. Values are means ± SD. vs. control group, * *p* < 0.05, ** *p* < 0.01

**Figure 2:**
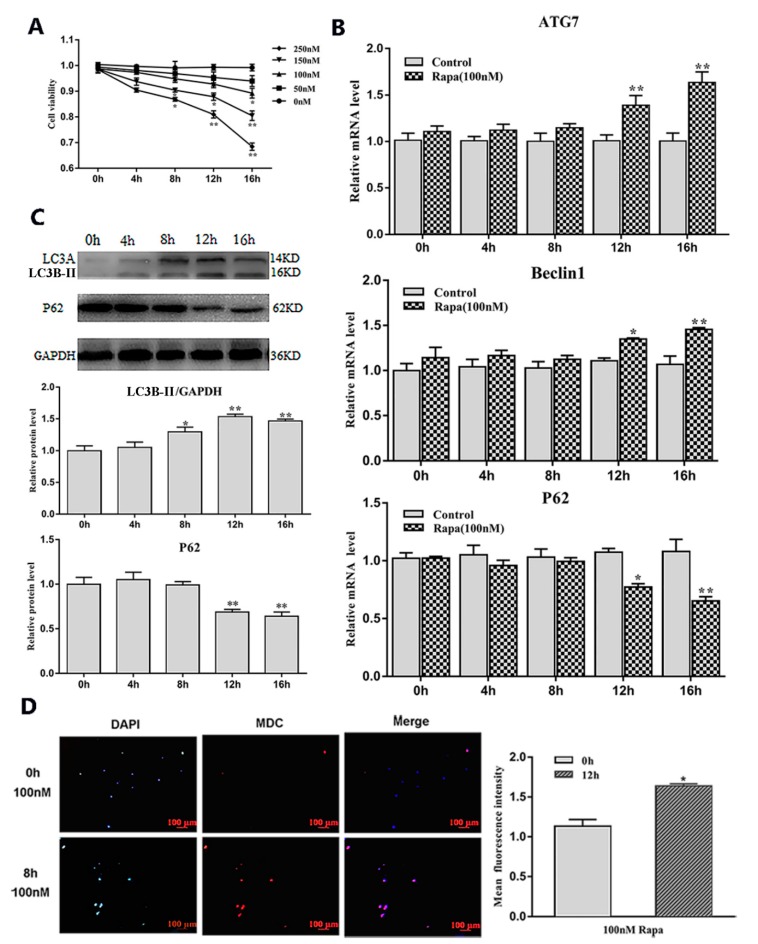
Adipocytes autophagy was induced by Rapa treatment. (**A**) Cell viability were detected by CCK8 (*n* = 6). (**B**) Relative mRNA level of autophagy-related genes with or without Rapa for different lengths of time (*n* = 6). (**C**) Protein level of autophagy-related genes with or without Rapa for different lengths of time (*n* = 6). (**D**) MDC and DAPI staining by 100 nM Rapa treatment for 12 h. Values are means ± SD. vs. control group, * *p* < 0.05, ** *p* < 0.01.

To study the effects of serum starvation and rapamycin (Rapa) treatment on adipocyte autophagy, we first induced 3T3-L1 preadipocytes to differentiate into mature adipocytes (Figure 1A–C). Then, we cultured mature adipocytes with serum-free medium and serum medium supplemented with a certain concentration of Rapa and selected the most appropriate culture time (8 h) and concentration (100 nM) (Figure 1D and Figure 2A). After the end of the culture, the RNA and protein were extracted for related detection. The results showed that serum starvation for 8 h and 100 nM Rapa treatment for 12 h did not change cell viability (Figure 1D and Figure 2A) (*p* > 0.05), but the expression of autophagy-related genes *Beclin1* and *ATG7* were significantly higher than the control group (*p* < 0.05) (Figure 1E and Figure 2B). A reliable marker of autophagy was the conversion of the ATG protein LC3 from a soluble form (LC3A) to a lipidized form (LC3B-II), which was stably associated with the membranes of autophagosomes. This conversion can be detected by measuring the accumulation of the LC3B-II formation. Western blot analysis showed an increase of LC3B-II upon serum starvation for 8 h and Rapa for 12 h treatment (*p* < 0.05) (Figure 1F and Figure 2C). It is well known that Monodansylcadaverine (MDC) accumulates specifically in autophagosomes or autophagic vacuoles (AV). Next, we examined the number of AV. These results showed that both serum starvation and Rapa treatment could increase the number of AVs (*p* < 0.05) (Figure 1G and Figure 2D). Therefore, we conclude that serum starvation and Rapa treatment can induce adipocyte autophagy.

Mark4 promoted serum starvation-induced autophagy via activating the AMPK signaling pathway and depressing AKT/ mTOR signaling pathways.

**Figure 3:**
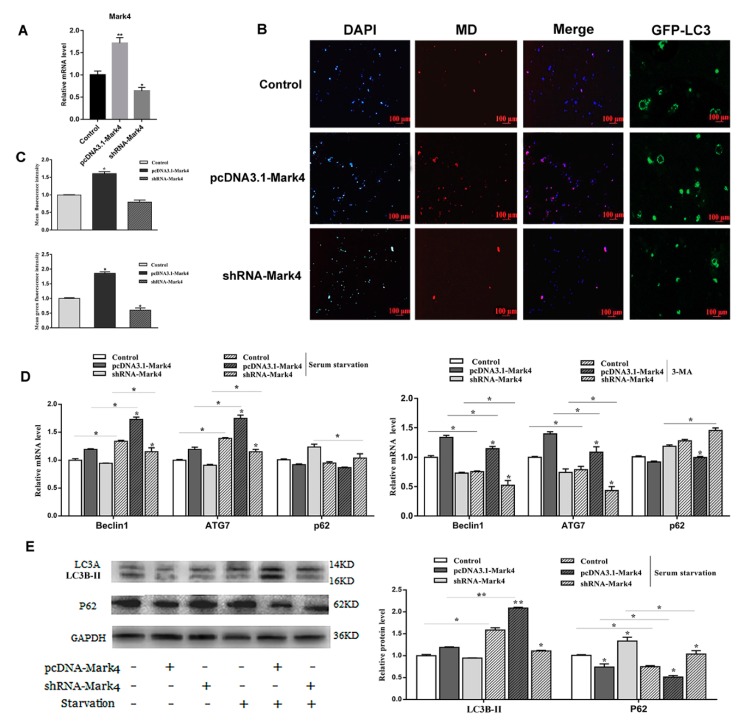
Microtubules affinity regulated kinase 4 (Mark4) promoted serum starvation-induced autophagy. (**A**) The adipocytes were pretreated with pcDNA-*Mark4* or shRNA-*Mark4* for 24 h, overexpression and interference efficiency of *Mark4* were detected (*n* = 6). (**B**) MDC and DAPI staining. Scale bar, 100 µm (*n* = 6). (**C**) Quantitative analysis of fluorescence intensity. (**D**) Relative mRNA level of autophagy-related genes. (**E**) Protein level of autophagy-related genes. Values are means ± SD. vs. control group, * *p* < 0.05, ** *p* < 0.01.

To further study the effect of Mark4 on autophagy, we constructed a *Mark4* overexpression and interference vector and tested the efficiency of overexpression and interference (*p* < 0.05) (Figure 3A). We then examined the occurrence of autophagy in adipocytes treated with or without *Mark4* plasmid vector. We found that the number of autophagic vesicles in the Mark4 group was increased compared with the control group (*p* < 0.05) (Figure 3B,C). The punctate appearance of endogenous LC3 confirmed the formation of autophagosomes (Figure 3B,C). Next, we detected the mRNA and protein expression of autophagy-related markers after 8 h of serum starvation. The mRNA levels of *Beclin1* and *ATG7* were significantly increased in Mark4 group compared with that of the control group (*p* < 0.05) (Figure 3D). Then, we studied the effect of Mark4 on autophagy by adding 3-methyl adenine (3-MA), an inhibitor of PI3K. The results showed that 3-MA pretreatment further inhibited the mRNA levels of Beclin1 and ATG7 (*p* < 0.05) (Figure 3D), whereas it was reversed in the Mark4 group. Consistently, we found that the LC3B-II lipidation (LC3B-II/LC3A ratios) and transcriptional levels were elevated, whereas autophagy substrate p62 level was lower in the Mark4-overexpressing group compared with the control group (*p* < 0.05) (Figure 3E).

**Figure 4:**
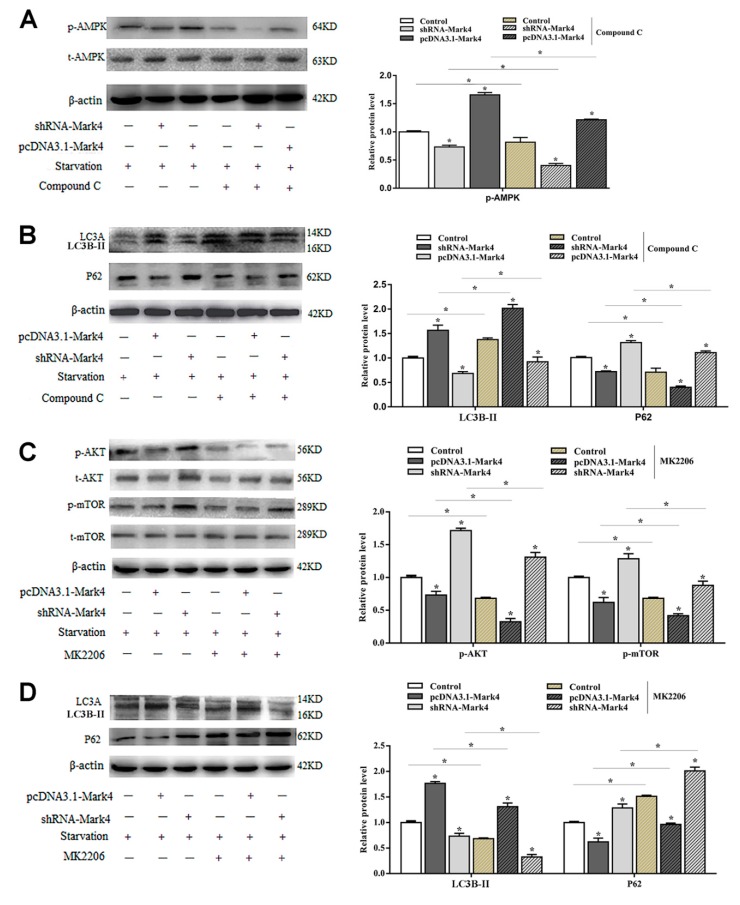
Mark4 promoted serum starvation-induced autophagy via AMP-activated protein kinase (AMPK)/AKT signaling pathways. Adipocytes were pretreated with pcDNA-*Mark4* or shRNA-*Mark4* or MK-2206 or Compound C. (**A**,**B**) Representative immunoblots and densitometric quantification for p-AMPK, LC3B-II, LC3A, and P62. (**C**,**D**) Representative immunoblots and densitometric quantification for p-Akt, p-mTOR, LC3B-II, LC3A, and P62. The level of total β-actin was determined as loading control. Values are means ± SD. vs. control group, * *p* < 0.05.

AMPK is a key regulator of energy metabolism-related pathways in the body, and its activation directly affects the body’s energy balance. Mark4 can negatively regulate the insulin signaling pathway by inhibiting AKT activation and reducing insulin sensitivity. These two signaling pathways are closely related to energy metabolism. To further characterize the underlying mechanisms for the regulation of Mark4 on serum starvation-induced adipocytes autophagy, we detected the AMPK and AKT signaling pathways. Overexpression of *Mark4* led to an upregulation of the ratio of phosphorylated AMPK to total AMPK (*p* < 0.05) (Figure 4A). The expression of autophagy protein LC3B-II was significantly increased, while the expression of autophagy inhibitor P62 was decreased. Compound C is an inhibitor of the AMPK signaling pathway. By adding compound C, the phosphorylation of AMPK and the formation of LC3B-II were reduced while the level of autophagy substrate p62 was increased (Figure 4A,B). The results of AKT pathway examination was opposite. The overexpression of *Mark4* significantly inhibited the phosphorylation of AKT and mTOR, accompanied by an increase in LC3B-II and a decrease in P62. After treating with the AKT inhibitor MK2206, the phosphorylation levels of AKT, mTOR, and the protein level of P62 were significantly reduced. The LC3B-II expression was increased (Figure 4C,D). These results indicated that Mark4 could regulate serum starvation-induced autophagy via the AMPK/AKT signal pathway.

### 2.2. Mark4 Promoted Rapa-Induced Autophagy via Inhibiting the mTOR Signaling Pathway

**Figure 5:**
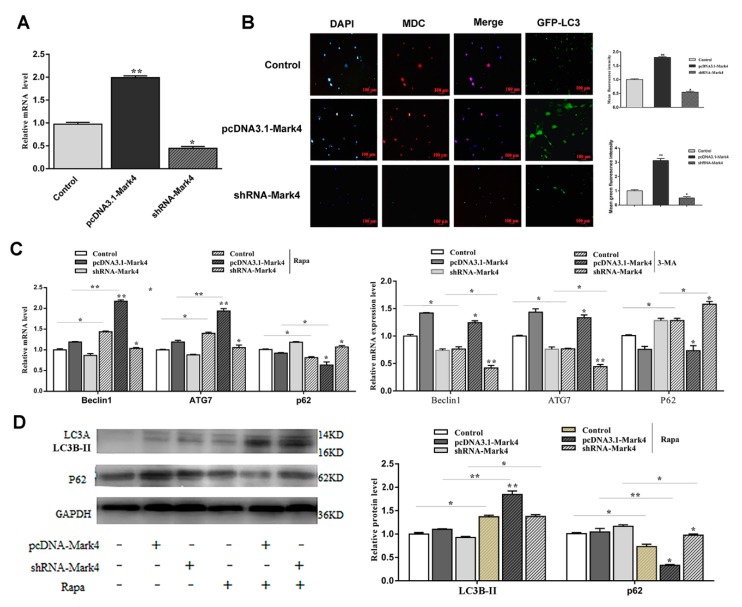
Mark4 promoted Rapa-induced autophagy. (**A**) The overexpression or interference efficiency of *Mark4* after the pretreatment of pcDNA-*Mark4* or shRNA-*Mark4* for 24 h (*n* = 6). (**B**) MDC staining and GFP-LC3 expression as indicated after transfection with Mark4 plasmid vector. Scale bar, 100 µm (*n* = 6). (**C**) Relative mRNA level of autophagy-related genes (**D**) Protein level of autophagy-related genes. Values are means ± SD. vs. control group, * *p* < 0.05, ** *p* < 0.01.

First, we detected the overexpression and interference efficiency of *Mark4* and confirmed that it met the requirements of the next stage of the experiment (*p* < 0.05) (Figure 5A). In this study, MDC staining was used to detect changes in the number of autophagic vesicles after transfection with *Mark4* plasmid vector. As shown in Figure 5B, the overexpression of *Mark4* increased the number of intracellular autophagic vesicles. The punctate appearance of endogenous LC3 also confirmed that Mark4 promoted the formation of autophagosomes (Figure 5B). Next, we investigated the mRNA and protein expression levels of autophagy-related markers after treating with 100 nM Rapa for 12 h. The results showed that mRNA levels of *Beclin1* and *ATG7* were significantly increased in the Mark4 group compared with the control group (*p* < 0.05) (Figure 5C). The addition of 3-MA further inhibited the mRNA levels of *Beclin1* and *ATG7* (*p* < 0.05) (Figure 5C). Consistently, we found that the LC3B-II lipidation and transcriptional levels were increased, whereas autophagy substrate p62 level was lower in the *Mark4*-overexpressing group compared with the control group (*p* < 0.05) (Figure 5D).

**Figure 6:**
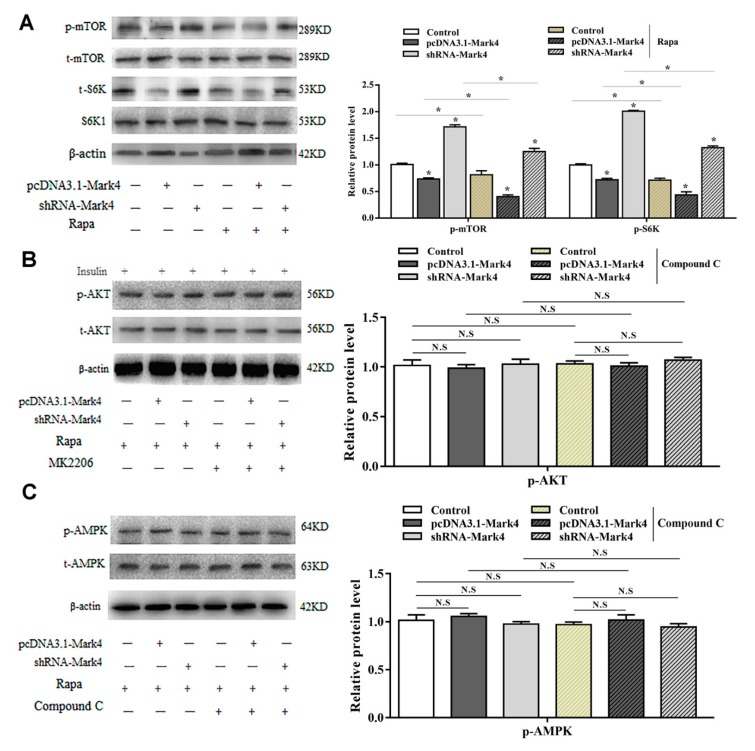
Mark4 promoted Rapa-induced autophagy via mTOR signaling pathway independent on AMPK/AKT signaling pathway. Adipocytes were pretreated with pcDNA-*Mark4* or shRNA-*Mark4* or Rapa or MK-2206 or Compound C. (**A**) Protein level of p-mTOR, t-mTOR, p-S6K, and S6K1 (*n* = 6). (**B**) Protein level of p-AKT and t-AKT (*n* = 6). (**C**) Protein level of p-AMPK and t-AMPK (*n* = 6). Values are means ± SD. vs. control group, * *p* < 0.05, N.S.

To further characterize the potential regulatory role of Mark4 in Rapa-induced adipocyte autophagy, we evaluated the mTOR pathway. By overexpression of *Mark4*, phosphorylated mTOR (Ser2448) and phosphorylated S6K1 (Thr389) were downregulated (Figure 6A). Interestingly, phosphorylated of AMPK and AKT were not changed through overexpression of *Mark4* (Figure 6B, C). In contrast, the mTOR-specific inhibitor Rapa markedly decreased the phosphorylation levels of mTOR and S6K1 (Figure 6A) while the overexpression of *Mark4* attenuated the effects of Rapa. These results indicated that Mark4 could regulate Rapa-induced autophagy via the mTOR signal pathway, independent of AMPK/AKT signal pathways.

### 2.3. Mark4 Decreased Browning of White Adipose Tissue via Promoting Autophagy

**Figure 7:**
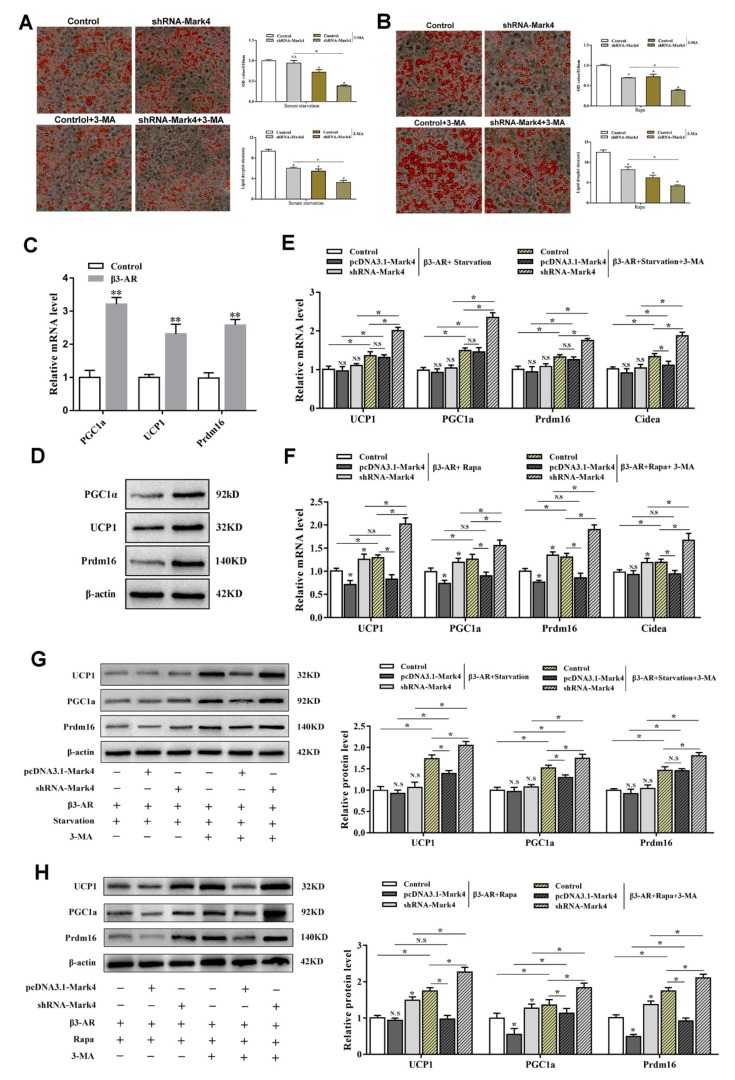
Mark4 decreased browning of white adipose tissue via promoting autophagy. (**A**) Oil red O staining after interference with *Mark4* and the addition of 3-methyl adenine (3-MA) under the condition of serum starvation. Scale bar, 100 µm (**B**) Oil red O staining after interference with *Mark4* and the addition of 3-MA under the treatment of Rapa. Scale bar, 100 µm. (**C**) Relative mRNA level of the marker genes of brown adipose tissue. (**D**) Protein level of the marker genes of brown adipose tissue. (**E**) Relative mRNA level of thermogenesis genes (*n* = 6). (**F**) Relative mRNA level of thermogenesis genes (*n* = 6). (**G**) Protein level of UCP1, PGC1a, and Prdm16. (**H**) Protein level of UCP1, PGC1a, and Prdm16. * *p* < 0.05, ** *p* < 0.01, N.S.

In order to further explore the potential connections among Mark4, autophagy, and browning of white adipose tissue, we designed the following experiments. Oil red O staining results showed that the knockdown of *Mark4* did not affect the number of lipid droplets on the basis of serum starvation treatment with smaller lipid droplets. After the 3-MA treatment, the number of lipid droplets significantly reduced, and the lipid droplets became much smaller (*p* < 0.05) (Figure 7A). By Rapa treatment, this change was more obvious (Figure 7B). In order to further explore the effect of Mark4 on the browning of white adipose tissue during starvation-induced autophagy, we first constructed the browning model by treating adipocytes with β3-adrenergic receptor agonist (β3-AR) while the β3-AR agonist is considered to be a classic reagent for building browning models [23]. The results showed that mRNA and protein expression of the marker genes of brown adipose tissue increased significantly after treating with β3-AR treatment (*p* < 0.01), proving that the browning model was successfully constructed (Figure 7C,D). Next, we examined the effect of Mark4 on the browning of white adipose tissue during autophagy in the case of β3-AR pretreatment. Results showed that serum starvation treatment alone did not affect the expression of UCP1, PGC1a, Prdm16, and Cidea (*p* > 0.05) (Figure 7E). By the 3-MA treatment, UCP1, PGC1a, Prdm16, and Cidea expression were significantly increased (*p* < 0.05) (Figure 7E). In the case of serum starvation and 3-MA co-treatment, the expression of UCP1, PGC1a, and Prdm16 did not change in the pcDNA3.1-*Mark4* group, but the expression of Cidea increased significantly (Figure 7E). After the interference of *Mark4*, the expressions of genes related to browning of white adipose tissue were further increased. Western blot results further confirmed these findings (Figure 7G). The detection results on the Rapa-induced autophagy model were consistent with those on the starvation-induced autophagy model. The expression of UCP1, PGC1a, Prdm16, and Cidea were increased significantly under the conditions of Rapa and 3-MA co-treatment. The trend was particularly obvious in the shRNA-*Mark4* group (Figure 7F,H). We guess that the reason for this result may be that 3-MA and shRNA-Mark4 have a superimposed effect on the inhibition of autophagy and thus significantly increase the expression of genes related to browning of white adipose tissue. Similarly, although the overexpression of *Mark4* significantly inhibited the expression of UCP1, PGC1a, and Prdm16 on Rapa-induced adipocyte autophagy, this effect was attenuated after giving 3-MA treatment (Figure 7F,H).

Cold stimulation and high-fat diet affect the expression of Mark4, autophagy-related genes, and thermogenesis genes.

**Figure 8:**
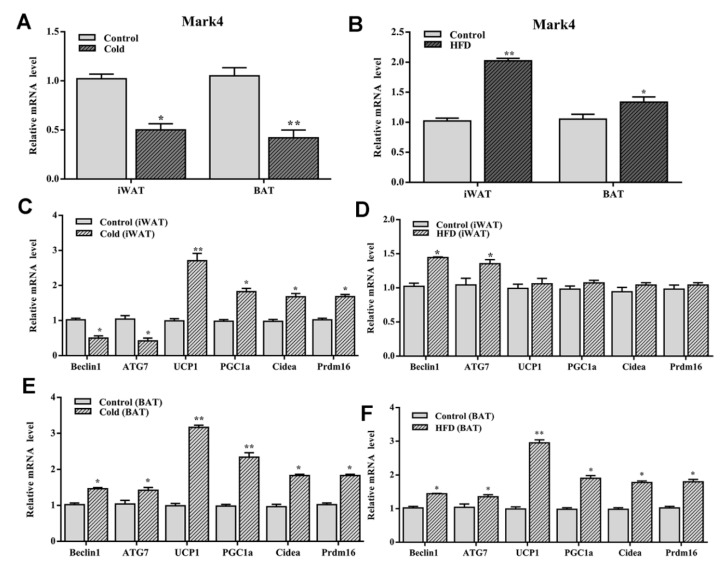
Cold stimulation and high-fat diet (HFD) influenced the expression of *Mark4*, autophagy-related genes and thermogenesis genes. (**A**,**B**) The expression level of *Mark4* on 4 °C environment and HFD. (**C**,**D**) Autophagy related genes and thermogenesis genes expression in white adipose tissue. (**E**,**F**) Autophagy related genes and thermogenesis genes expression in brown adipose tissue. * *p* < 0.05, ** *p* < 0.01.

To clarify the effects of cold stimulation on autophagy and browning of white adipose tissue, the expression of Mark4, autophagy-related genes, and thermogenic genes in adipose tissue were measured after the cold stimulation. As shown in Figure 8A, the expression level of *Mark4* was significantly reduced in both WAT and BAT (*p* < 0.05). Autophagy-related genes *Beclin1* and *ATG7* were downregulated, but the thermogenic genes *UCP1, PGC1a, Prd16*, and *Cidea* were significantly increased in WAT (*p* < 0.05) (Figure 8C). However, both autophagy-related genes and thermogenesis genes were upregulated significantly in BAT (Figure 8E). Based on the above results, we concluded that Mark4 regulated autophagy of WAT and BAT by different mechanisms.

To explore the role of high-fat diet in Mark4, autophagy, and thermogenesis, we established an obese mouse model using a high-fat diet. The results showed that *Mark4* expression levels were upregulated in both WAT and BAT (Figure 8B). Though autophagy-related genes *Beclin1* and *ATG7* were increased, thermogenesis genes *UCP1, PGC1a, Prdm16*, and *Cidea* had no change in WAT (*p* > 0.05) (Figure 8D). However, both autophagy-related genes and thermogenesis genes were upregulated significantly in BAT (Figure 8F).

## 3. Discussion

Obesity is characteristic of adipose tissue occupying too much of the whole body. The size of adipose tissue is determined by the number and volume of adipocytes, which depends on the intracellular triglyceride synthesis and catabolism efficiency. Recently, it was hypothesized that excess lipid accumulation directly contributes to metabolic syndrome in adipose tissue, which has generated increased interest. Mark4 is associated with the basal body and was required for the initiation of axoneme extension after the docking of ciliary vesicles to the mother centriole [24]. Our previous research showed increased appetite, activity, and metabolic rate in *Mark4* knockout mice against obesity induced by high-fat diet. AKT and AMPK signaling pathways were activated to alleviate insulin resistance and maintain glucose homeostasis [9]. We further determined that Mark4 promoted adipogenesis by activating JNK and p38MAPK signaling pathways. The activation of the JNK signaling pathway can trigger adipocyte apoptosis [10]. In addition, there are reports that Mark4 promotes oxidative stress and inflammation via binding to PPARγ and activating the NF-κB pathway in mice adipocytes [11]. These data establish novel regulation roles of Mark4 in body metabolic balance.

Autophagy is a major cellular degradation pathway as it can remove the excess or dysfunctional proteins and organelles to maintain cellular homeostasis [25]. Studies have confirmed that the inhibition of adipose-specific autophagy leads to the increased number of adipocytes with features of brown adipocytes. This facilitated fatty acid oxidation and increased insulin sensitivity both in vitro and in vivo [26]. We speculate that Mark4, autophagy, and browning of white adipose tissue have certain linkages, whether Mark4 can influence autophagy has not been reported. Although serum starvation and Rapa-induced autophagy are classical methods, previous research has focused on macrophages, tumor cells, and cancer cells described above. There is little research on adipocyte autophagy. Therefore, we established a serum starvation and Rapa-induced autophagy model in adipocytes. In this study, overexpression of *Mark4* promoted the expression of autophagy positive regulator Beclin1, ATG7, and LC3B-ll while inhibited the expression of negative regulators P62 regardless of treatment with serum starvation or Rapa. These results showed that Mark4 had a positive regulation on cell autophagy by serum starvation or Rapa treatment in adipocytes.

The yeast homologue ULK1-ATG1 plays an important role in activating downstream components in autophagy. It may negatively regulate the ULK1 complex through the mTOR signaling pathway under the condition of nutrition sufficient [27,28]. MTORC1 dissociates from lysosomes when cells face starvation or lack of amino acids. This leads to a decrease in the phosphorylation levels of ULK1 and ATG13 and activates autophagy [29,30]. In this study we found the overexpression of *Mark4* improved the phosphorylation of AMPK after treating with serum starvation but inhibited AKT phosphorylation level. On the contrary, the knockdown of *Mark4* repressed AMPK phosphorylation level but increased AKT phosphorylation level in adipocytes. This effect was attenuated by Compound C and MK-2206 which are highly specific inhibitors of AMPK and AKT signaling pathways. Furthermore, we also found that Mark4 promoted Rapa-induced autophagy via inhibiting the mTOR signaling pathway. These findings confirmed that AMPK/AKT/mTOR signaling pathways were necessary for Mark4 function. Overexpression of *Mark4* could promote adipocyte autophagy by regulating AMPK/AKT/mTOR pathways.

**Figure 9:**
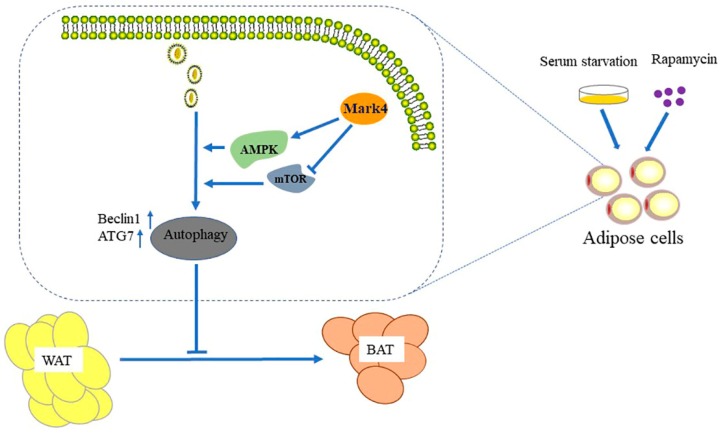
Mark4 inhibited the browning of white adipose tissue by promoting adipocytes autophagy.

White adipose tissue (WAT) plays a central role in the regulation of energy balance and acts as a secretory/endocrine organ that mediates numerous physiological and pathological processes [31]. Brown adipose tissue (BAT) has the capacity of modulating energy balance through the dissipation of energy in response to cold and excess feeding [32,33]. In cold-acclimated mice, the number of brown adipocytes increases in white adipose tissue [34,35]. This phenomenon is often referred to as “browning” of WAT. Additionally, recent data seem to support that white and brown adipocytes can undergo bidirectional intercom-version [36]. It is worth noting that its abundance is inversely proportional to the body mass index [37]. It is a classic method to study browning by building a model, while β3-AR agonist has been considered as a classic reagent for building a browning model [38]. In our studies, the overexpression of *Mark4* decreased the expression of thermogenesis genes via promoting autophagy, thereby inhibiting the browning of white adipose tissue (Figure 9). The interference of *Mark4* made lipid droplets smaller and fewer, which was a characteristic of WAT browning. For this reason, browning of WAT has recently emerged as a promising alternative therapeutic strategy to curb obesity and related disorders [39,40,41]. This conversion between WAT and BAT also provides new ideas for improving meat quality in animal husbandry production.

## 4. Materials and Methods

### 4.1. Animal Experiment

Two-week-old Kunming male mice were purchased from the Laboratory Animal Center of the Fourth Military Medical University (Xi’an, China). All mice experiments were carried out in accordance with applicable guidelines and regulations (T/NWSUAF, 235-2014, 2014-05-09) approved by the Animal Ethics Committee of Northwest A&F University (Yangling, China) (October, 2017~December, 2017). They were allowed ad libitum access to water and standard chow laboratory diet for the first 2 weeks to allow them to adjust to the new environment. Mice were subsequently randomly assigned to four groups: a high-fat diet fed group (87.5% chow diet + 10% lard +2% cholesterol +0.5% bile salt; Animal Center of the Fourth Military Medical University) or a chow diet fed group (Animal Center of the Fourth Military Medical University) for the next 6 weeks; a cold-stimulating group (4 °C for 24 h), a normal temperature group (30 °C for 24 h). Inguinal white adipose tissue and scapular brown adipose tissue were harvested. The animal room was maintained under controlled conditions of temperature at 25 ± 1 °C, humidity at 55 ± 5%, and a 12-h light/12-dark cycle.

### 4.2. Cell Culture

Mouse 3T3-L1 pre-adipocytes were inoculated at a concentration of 1 × 10^6^/mL and cultured in Dulbecco’s modified Eagle’s medium (DMEM, St. Louis, MO, USA) high glucose (Gibco, Carlsbad, CA, USA) with 10% fetal bovine serum (Sigma, St. Louis, MO, USA) at 37 °C and 5% CO_2_. Confluent cells were differentiated at day 0 in DMEM high glucose with 10% FBS, 10 µg/mL bovine insulin (Sigma, St. Louis, MO, USA), 1 µM dexamethasone (Sigma, St. Louis, MO, USA), 0.5 mM isobutyl-1-methylxanthine (Sigma, St. Louis, MO, USA). On day 2, media was changed with DMEM high glucose, 10% FBS, and 10 µg/mL bovine insulin. Day 4 and afterwards, cells were cultured in DMEM high glucose plus 10% FBS.

Epididymal white adipose tissues of mice were harvested, visible fibers and blood vessels were removed, and the adipose tissue was washed three times with PBS buffer containing 200 U/mL penicillin (Sigma, St. Louis, MO, USA) and 200 U/mL streptomycin (Sigma, St. Louis, MO, USA). Then the adipose tissue was minced into fine sections (1 mm^3^) with scissors and incubated in 10 mL of digestion buffer containing DMEM/F-12 (Gibco), 100 mM HEPES (Sigma, St. Louis, MO, USA), 1.5% BSA (Sigma, St. Louis, MO, USA), and 2 mg/mL type I collagenase (Sigma, St. Louis, MO, USA) for 50 min at 37 °C in a water bath. After the incubation, growth medium (DMEM/F-12 (50:50)), 10% fetal bovine serum (Sigma, St. Louis, MO, USA), 100 U/mL penicillin, and 100 U/mL streptomycin were added to the digestion flask. Flask contents were mixed and filtered through nylon screens with 250 and 20 μm mesh openings to remove undigested tissue and large cell aggregates. The filtered cells were centrifuged at 300× *g* for 7 min at room temperature to separate floating adipocytes from cell pellets. Isolated cell pellets were suspended in DMEM/F12 (Invitrogen, Carlsbad, CA, USA). Finally, cells were seeded into 35-mm primary culture dishes at a density of 8 × 10^4^ cells/dish and incubated at 37 °C under a humidified atmosphere of 5% CO_2_ and 95% air until confluence. The medium was changed every other day [42].

### 4.3. Cell Viability Assay

Cell viability was measured using by CCK-8 (Vazyme, Nanjing, China) assay. The transfected cells were seeded in 96-well plates at a density of 5 × 10^3^ and cultured for 12 h. Then, 10 μL CCK-8 solution was added into each well and incubated for 1 h at 37 °C. Absorbance was quantified at 450 nm by Vector 5 (Bio-TechInstruments, Winooski, VT, USA).

### 4.4. GFP-LC3 Analysis and Subcellular Localization

Cells were transfected with GFP-LC3 plasmid by using X-treme GENE HP Reagent (Roche, Basel, Switzerland) according to the manufacturer’s instructions. After 48 h transfection, cells were washed with OptiMEM I (Invitrogen, Carlsbad CA, USA, 51985042) and subjected to staining. The cells were stained with LysoTracker^®^ Green DND probe (Thermo Scientific, Carlsbad, CA, USA, L7526) as recommended by the manufacturer. The formation of GFP-LC3 punctate and Tracker fluorescence were visualized and analyzed using Cytation3 Cell Imaging Multi-Mode Reader (BioTek, Winooski, VT, USA) [20]. The cells transfected with the GFP-LC3 plasmid were continuously passed to five generations, and the fluorescence expression of the 1st, 3rd, and 5th generation cells was detected to determine the efficiency and stability of transfection (results are not shown).

### 4.5. Transfection of Adipocytes with Plasmids

Mark4 forced expression plasmid vector HA-Mark4 was kept in our lab. shRNA sequence against Mark4 was contrived and synthesized by Genepharma Company (Shanghai, China) using pGPU6/Neo shRNA expression vectors named sh1-Mark4, sh2-Mark4, and sh3-Mark4. Then by transfection efficiency detection, the optimal shRNA of Mark4 was chosen and named sh-Mark4. Plasmids vectors used as control vectors were pcDNA3.1-vector and negative-shRNA. To exclude off-target effects of shRNA treatment, we used the other two Mark4 shRNAs, sh1-Mark4 and sh3-Mark4 which targeted different sequences of Mark4 mRNA compared with shRNA-Mark45. Mark4 DA (dead mutant) was made as described previously [7]. In HA-Mark4 DA group, Mark4 protein was translated inactively with specific amino acid mutations called dead mutant. Then, 2 μg interference or expression plasmids DNA were mixed with 2 μL X-treme GENE HP Reagent (Roche, Switzerland) and Opti-MEMI media (Invitrogen, California, USA) and then added into the culture dish for 24 or 48 h according to the protocol [11].

### 4.6. MDC Staining Assessment

The MDC assay was performed in each sample by using an in situ cell autophagy detection kit (keyGEN BioTECH, Nanjing, China). The adipocytes were grown to 60% confluence in 60-mm culture dishes and Mark4 reconstructed plasmids were transfected into cells with X-treme GENE HP. After differentiation for 4 days, adipocytes were trypsinized and suspended at 1 × 10^6^ cells in 300 μL 1×Wash Buffer. To the cell suspension, 90 μL cell suspension was drawn into a new eppendorf tube, then cells were stained by MDC fluorochrome for 30 min at room temperature. Cells were washed by 1×Wash Buffer three times and then stained by DAPI for 10 min at room temperature. At last cells were observed and imaged by inverted fluorescent microscopy. The area of cells stained with MDC was measured by Image-Pro Plus analyzer (Media Cybernetics, Inc, Rockville, MD, USA).

### 4.7. Oil Red O Staining

Cells were washed three times in PBS buffer and then fixed in 10% (*v*/*v*) formaldehyde for 30 min. The fixed cells were then washed three times in PBS and stained with a working solution of Oil Red O for 15 min at room temperature. Cells were washed with cooling PBS two times and image observation was taken with a Nikon TE2000-U Fluorescence Microscope (Tokyo, Japan). Then Oil Red O was extracted by 100% avantin for colorimetric analysis at 510 nm.

### 4.8. Real-Time Quantitative PCR Analysis

Total RNA was extracted with TRIpure Reagent kit (Takara, Dalian, China) and 500 ng of total RNA was reverse transcribed using the Roche reverse transcriptase kit (Roche, Shanghai, China). Primers for Mark4, Beclin1, ATG7, P62, UCP1, PGC1α, Prdm16, Cidea, GAPDH, and β-actin were synthesized by Shanghai Sangon Ltd. (Shanghai, China). GAPDH and β-actin were used as internal control in PCR amplification. Quantitative PCR was performed in 25 μL reactions containing specific primers and SYBR Premix EX Taq (Takara, Dalian, China). The levels of mRNAs were normalized to β-actin. The expression of genes was analyzed by method of 2^−∆∆Ct^.

### 4.9. Protein Extraction and Western Blot Analysis

Cells were lysed in RIPA buffer for 40 min at 4 °C centrifuge. Removing insoluble material by centrifugation at 12,000× *g* for 15 min at 4 °C, and the supernatants were used to assay protein levels. Protein samples (50 μg) were separated by electrophoresis on 12% and 5% SDS-PAGE gels using slab gel apparatus and then transferred to PVDF nitrocellulose membranes (Millipore, Boston, MA, USA) blocked with 5% Skim Milk Powder/Tween 20/TBST at room temperature for 2 h. Primer antibodies against Mark4 and GAPDH were purchased from Bioworld (MN, USA). Antibodies against LC3B-ll and LC3l, P62, UCP1, PGC1α, Prdm16, and Cidea were from Abcam (Cambridge, MA, USA). Additionally, antibodies against AMPK, phospho-AMPK, AKT, phospho-AKT, mTOR, and phospho-mTOR were from Cell Signaling (Danvers, MA, USA). Membranes were incubated with primary antibodies at 4 °C overnight and then incubated with the appropriate HRP-conjugated secondary antibodies (Boaoshen, China) for 2 h at room temperature. Proteins were visualized using chemiluminescent peroxidase substrate (Millipore, Boston, MA, USA), and then the blots were quantified using ChemiDoc XRS system (Bio-Rad, Shanghai, China) and quantitative analysis of immune-blotted bands was performed using Quality One software (Bio-Rad).

### 4.10. Drug Treatment in Adipocytes

Rapa, 3-MA, Compound C and MK2206 were purchased from Sellcek (St. Louis, MO, USA). Rapaworking solution (100 nM for 12 h), 3-MA working solution (5 mM for 4 h), Compound C (10 µM for 12 h), and MK2206 working solution (30 µM for 12 h) were prepared to treat cells before plasmid transfection. Insulin was purchased from Sigma (Shanghai, China), Insulin working solution (10 µM).

### 4.11. Statistical Analysis

Statistical calculations were performed with SAS v8.0 (SAS Institute, Cary, NC, USA). Statistical significance was determined using the one-way ANOVA test. Comparisons among individual means were made by Fisher’s least significant difference (LSD) post hoc test after ANOVA. Data are presented as mean ± SD; *p* < 0.05 was considered to be significant.

## Abbreviations

AMPK	AMP-activated protein kinase
Atf4	Activating transcription factor 4
Atg5	Autophagy-related gene 5
Atg7	Autophagy-related gene 7
AV	Autophagic vacuoles
BAT	Brown adipose tissue
CCK8	Cholecystokinin-8
ER	Endoplasmic reticulum
Fgf21	Fibroblast growth factor 21
HFD	High-fat diet
JNK	Jun N-terminal kinase
LC3A	Microtubule-associated protein light chain 3
LC3B	Microtubule associated protein 1 light chain 3β
MAPK	Mitogen-activated protein kinase
MAPs	Microtubule associated protein
MARKs	Microtubule associated protein serine/threonine kinase
Mark2	Microtubules affinity regulated kinase 2
Mark3	Microtubules affinity regulated kinase 3
Mark4	Microtubules affinity regulated kinase 4
MDC	Monodansylcadaverine
MR	Mineralocorticoid receptor
NF-κB	Nuclear factor-kappa B
PKB/AKT	Protein kinase B
PPARγ	Peroxisome proliferator-activated receptor gamma
Rapa	Rapamycin
WAT	White adipose tissue
β3-AR	β3-adrenergic receptor
3-MA	3-methyl adenine
